# Intestinal Intussusception in Adults: A Systematic Review

**DOI:** 10.1002/wjs.70055

**Published:** 2025-08-19

**Authors:** Sidney Heersche, Jeanne Hirt, Fabio Butti, Martin Hübner, Dieter Hahnloser, Gaëtan‐Romain Joliat, Fabian Grass

**Affiliations:** ^1^ Department of Visceral Surgery Lausanne University Hospital CHUV Lausanne Switzerland; ^2^ Faculty of Biology and Medicine University of Lausanne (Unil) Lausanne Switzerland

**Keywords:** intussusception, invagination, resection, surgery, treatment

## Abstract

**Background:**

Management of intestinal intussusception *remains* controversial with regards to conservative versus operative management, as well as choice of surgical procedure.

**Methods:**

A systematic review on PubMed/MEDLINE, Web of Science, Google Scholar, SCOPUS/EMBASE, and the Cochrane Library was performed. Articles published between 2004 and 2024 were collected. Case reports and case series < 20 patients were not considered.

**Results:**

After screening of 2286 articles, 37 studies totaling 2330 patients were included. All studies were retrospective case series except for one prospective case series and one prospective and retrospective case series. There were 20% of colocolic intussusceptions, 21% of ileocolic intussusceptions, and 65% of enteric (small bowel) intussusceptions. A radiological lead point of the intussusception was described in 31% of patients. Intussusceptions were idiopathic in 39%, and caused by a pathological lead point in 58% of cases. Underlying malignancy was present 25% of cases and was more frequent in case of colocolic intussusceptions (68%), versus 9% in small bowel intussusceptions, and 46% of ileocolic intussusceptions. Operative management was performed in 49% of intussusceptions, mainly through open access (72%). Based on these findings, a comprehensive algorithm is provided to guide treatment decisions in adult patients with intussusception.

**Conclusion:**

Data regarding surgical management of the intussusception were heterogenous and poorly reported. In case of colic intussusception, a high degree of suspicion for an underlying malignancy is warranted, and in that regard, an oncological surgical resection without reduction is recommended. Enteric intussusception should be managed according to length, clinical presentation, and interoperative findings of lead points.

## Graphical abstract

Management of intestinal intussusception remains controversial with regards to conservative versus operative management, as well as choice of surgical procedure. A systematic review on PubMed/MEDLINE, Web of Science, Google Scholar, SCOPUS/EMBASE, and the Cochrane Library was performed. Articles published between 2004 and 2024 were collected. Case reports and case series < 20 patients were not considered. After screening of 2286 articles, 37 studies totaling 2330 patients were included. All studies were retrospective case series except for one prospective case series and one prospective and retrospective case series.

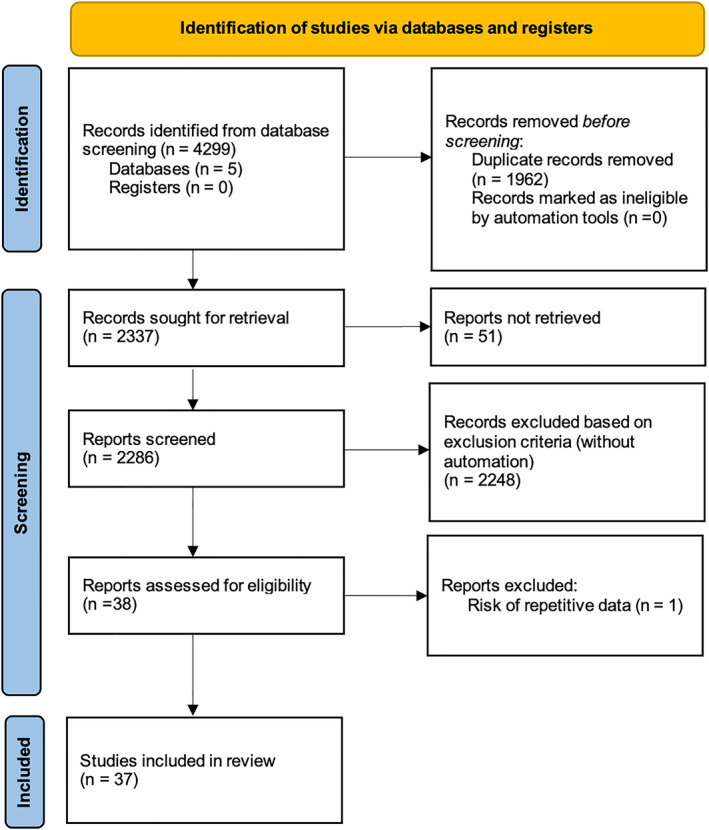

## 1 Introduction

Intussusception is defined as the telescoping or invagination of one segment of the gastrointestinal tract into an adjacent segment. This condition can lead to various complications, such as bowel obstruction, ischemia, and even perforation. It is most commonly observed in children, where it is typically primary and benign, often resolving with nonoperative reduction using a water‐soluble contrast enema [1]. In contrast, intussusception is rare in adults, accounting for approximately 5% of all cases of intussusception and accounting for approximately 1%–5% of intestinal obstructions [2]. Pediatric intussusception is characterized by the classic triad of abdominal pain, a palpable mass, and ‘red currant jelly' stool, although this triad is present in only a minority of cases [3]. In adults, diagnosis is more challenging due to nonspecific symptoms, which can be acute or chronic and often require further diagnostic work‐up, such as imaging or endoscopy. Whereas nonoperative management represents the initial treatment strategy in pediatric intussusceptions; adult intussusceptions involve a lead point in up to 92% of patients, potentially related to a malignant tumor and therefore requiring different management [4, 5]. Whereas surgery is often required to determine the underlying etiology, the optimal surgical strategy remains a subject of debate. Although surgical reduction may prevent unnecessary bowel resection, it carries risks, including intraluminal seeding or venous tumor dissemination in cases of malignancy, or spillage in cases of perforation [6, 7]. Currently, there are no established guidelines for the surgical management of intussusception in adult patients, with most available data derived from individual institutional case series.

With advancements in technology and the widespread use of CT‐scans in both emergency and elective settings, the detection rate of incidental or small intussusceptions is increasing. This has further fueled debate concerning their treatment and the possibility of conservative management.

For these reasons, the primary objectives of this study were to conduct a systematic review of the literature on adult intussusception and secondly to propose a management algorithm to guide surgeons in their daily practice.

## 2 Methods

### 2.1 Literature Search Strategy

A systematic search of the literature was performed by two authors independently (SH and JH; last date of assessment: August 2024). In case of disagreement or doubt regarding inclusion, two senior authors (GRJ and FG) decided whether the article fulfilled the eligibility criteria. This study followed the recommendations of the Supporting Information S1: PRISMA guidelines for reporting and the AMSTAR guidelines.

Articles were searched on PubMed/MEDLINE, Web of Science, Google Scholar, SCOPUS/Embase, and the Cochrane Library. Pertinent references and electronic links were hand searched. The full search strategy, including medical subject heading (MeSH) terms, is available as a Supporting Information S2: online appendix. References of the included articles were browsed in order not to miss potentially relevant studies (cross‐referencing). All studies of interest were obtained as full‐text articles. Reviews and publications not reporting on any of the outcomes of interest were excluded. Ongoing research was assessed through clinical trial registries (ClinicalTrials.gov and EU Clinical Trials Register).

### 2.2 Eligibility Criteria

Inclusion criteria were studies with 20 patients or more who had small bowel, ileocolic, or colic intussusception and studies published from 2004 until June 2024. All study types were considered except case reports, case series < 20 patients, letters to the editor, conference abstracts, or commentaries. Articles that included patients with intussusception after surgery, such as bariatric surgery or colorectal surgery, were excluded. Only articles in English were considered. Quality of selected articles was assessed using the JBI critical appraisal tool for case series studies in order to assess risk of bias [8].

### 2.3 Extraction of Data and Outcomes of Interest

Author names, publication year, country, study type (retrospective or prospective cohort), number of patients, age, sex, symptoms at initial presentation, acute (< 14 days) or chronic presentation, diagnostic means (CT‐scan, ultrasound, and endoscopy), intussusception location (enteric, ileocolic, or colic), lead point (on imaging or pathological report), presence of malignancy, and treatments (conservative, surgical access, bowel reduction, segmental resection, and oncological resection) were extracted from each included article, stored in a a priori structured database and collected in tables according to the following subsections: study selection and patient characteristics, diagnosis, etiology, and management.

The primary outcome of interest was the etiology causing the intussusception. Secondary outcomes were the performed treatment (conservative, surgical, type of surgical treatment/reduction, or resection), surgical access, and diagnostic procedure.

### 2.4 Statistics

All statistical analyses were performed using SPSS 29.0 for Mac OS X. Meta‐analysis was not performed because of often poorly reported data. Descriptive statistics were used to estimate an overall tendency and to summarize the included data. Discrete variables were summarized using pooled proportions, weighted means, and percentages. Effect measures were not performed due to the qualitative nature of reported outcomes.

No ethical approval was necessary as no patient data were included and only published data were collected.

### 2.5 Algorithm

Based on results from the selected articles, an algorithm for the management of the adult patient with intussusception was created.

## 3 Results

### 3.1 Study Selection and Patient Characteristics

**FIGURE 1:**
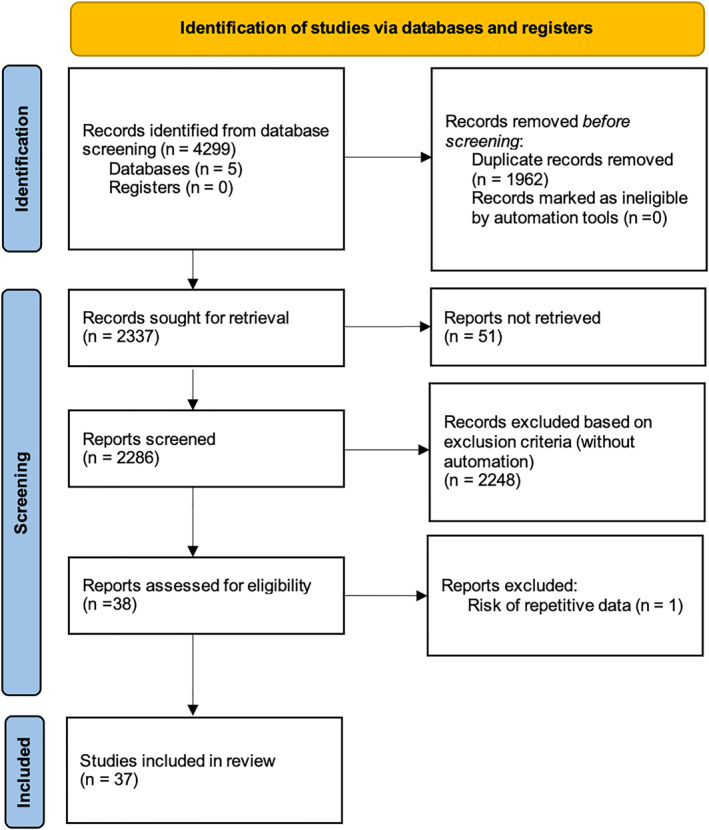
Flowchart.

The literature search found 2337 articles eligible for screening. Based on eligibility criteria, 37 articles remained for analysis, including a total of 2330 patients. The screening process is depicted in Figure 1. All studies were retrospective case series except for one prospective case series [9] and one prospective and retrospective case series [10]. Quality assessment for all studies with the JBI critical appraisal tool for case series studies [8] was performed, with a median score of 9/10 points. The complete assessment is available as Supporting Information S2. Reasons for article exclusion are depicted in the study flowchart (Figure 1). Details of the final article selection (included studies) as well as patient characteristics are shown in Table 1.

**TABLE 1:** Study characteristics.

First author	Year	Study type	Country	Patients *n*	Intussusceptions *n*	Age (mean)	Men/women	Surgical *n* (%)	Conservative *n* (%)
Dong [11]	2023	Retrospective c.s.	China	88	91	60	58/30	91 (100)	NA
Álvarez‐Bautista [12]	2023	Retrospective c.s.	Mexico	28	28	46	9/19	25 (89)	3 (11)
Sun [13]	2022	Retrospective c.s.	China	51	51	58	21/30	51 (100)	NA
Hu [14]	2021	Retrospective c.s.	China	71	91	46	44/27	91 (100)	NA
Neymark [15]	2021	Retrospective c.s.	Israel	76	76	43	NA	49 (64)	27 (36)
Dollinger [16]	2021	Retrospective c.s.	Germany	75	103	45	56/19	12 (12)	91 (88)
Duc [17]	2021	Retrospective c.s.	Vietnam	96	99	53	55/41	85 (86)	14 (14)
González‐Carreró [18]	2021	Retrospective c.s.	Spain	21	21	57	9/12	17 (81)	4 (19)
Kim [19]	2021	Retrospective c.s.	Republic of Korea	28	28	NA	10/18	28 (100)	NA
Kang [20]	2020	Retrospective c.s.	Republic of Korea	71	78	51	44/27	78 (100)	NA
Siow [21]	2019	Retrospective c.s.	Malaysia	37	37	49	19/18	37 (100)	NA
Kim [22]	2018	Retrospective c.s.	Republic of Korea	77	77	51	38/39	77 (100)	NA
De Clerck [6]	2016	Retrospective c.s.	Belgium	43	43	47	15/28	31 (72)	12 (28)
Honjo [23]	2015	Retrospective c.s.	Japan	44	44	70	20/24	41 (93)	3 (11)
Amr [24]	2015	Retrospective c.s.	USA	318	318	51	134/184	192 (60)	126 (40)
Somma [25]	2014	Retrospective c.s.	Italy	47	47	49	22/25	32 (68)	15 (32)
Varban [26]	2013	Retrospective c.s.	USA	44	44	51	22/22	44 (100)	NA
Cakir [27]	2013	Retrospective c.s.	Turkey	47	47	49	23/24	47 (100)	NA
Lindor [28]	2012	Retrospective c.s.	USA	148	148	48	64/84	77 (52)	71 (48)
Gupta [29]	2011	Retrospective c.s.	India	27	28	37	NA	28 (100)	NA
Gupta [30]	2011	Retrospective c.s.	Nepal	38	38	50	24/14	38 (100)	NA
Gollub [31]	2011	Retrospective c.s.	USA	33	34	63	15/18	23 (68)	11 (32)
Tabrizian [32]	2010	Retrospective c.s.	USA	80	80	45	34/46	18 (23)	62 (77)
Sundaram [33]	2009	Retrospective c.s.	USA	118	136	44	64/54	8 (6)	128 (94)
Yakan [34]	2009	Retrospective c.s.	Turkey	20	20	48	9/11	20 (100)	NA
Wang [35]	2009	Retrospective c.s.	China	39	40	44	17/21	39 (98)	1 (2)
Olasky [36]	2009	Retrospective c.s.	USA	23	23	44	16/7	11 (48)	12 (52)
Ahn [37]	2009	Retrospective c.s.	Republic of Korea	42	42	50	20/22	42 (100)	NA
Chang [38]	2009	Retrospective c.s.	Taiwan	46	46	58	28/18	44 (96)	2 (4)
Tresoldi [39]	2008	Retrospective c.s.	Italy	93	93	49	53/40	20 (22)	73 (88)
Rea [40]	2007	Retrospective c.s.	USA	170	186	41	80/90	46 (25)	140 (75)
Park [41]	2007	Retrospective c.s.	Republic of Korea	24	24	48	14/10	24 (100)	NA
Zubaidi [2]	2006	Retrospective c.s.	Canada	22	22	57	9/13	21 (95)	1 (5)
Maconi [42]	2006	Retrospective + prospective c.s.	Italy	32	33	38	19/13	3 (10)	30 (90)
Goh [43]	2006	Retrospective c.s.	Singapore	60	60	58	34/26	60 (100)	NA
El Fortia [44]	2006	Prospective c.s.	Libya	30	30	35	21/0	30 (100)	NA
Sandrasegaran [45]	2004	Retrospective c.s.	USA	24	26	42	14/10	1 (4)	25 (96)

Abbreviations: c.s., case series; NA, not available.

### 3.2 Diagnosis

In total, 2330 patients presented with 2432 intussusceptions. Eighteen studies used CT for diagnosis of intussusception. Other diagnostic modalities throughout the remaining studies (*n* = 19) were ultrasound, colonoscopy, and plain abdominal films. Based on these studies, the correct pre‐operative diagnosis of intussusception (confirmed by operative management) for CT and ultrasound was achieved in 82% (based on 17 studies) and 54% (based on 13 studies), respectively. Colonic intussusceptions were accurately diagnosed in 74% (based on 9 studies) of patients who underwent a colonoscopy. A radiological lead point of the intussusception was described in 31% of patients (291/953 patients, based on 16 studies). There were 20% of colocolic intussusceptions (442/2259, based on 33 studies), 21% of ileocolic intussusceptions (410/1906, based on 27 studies), and 65% of enteric (small bowel) intussusceptions (1540/2368, based on 35 studies).

### 3.3 Etiology of the Intussusception

Intussusceptions were classified as idiopathic when managed conservatively without a lead point visualized on imaging studies, or when no pathological lead point or intussusception was found during surgical exploration. Idiopathic intussusceptions accounted for 39% of the cases (789/1991 patients based on 29 articles).

In patients who underwent operative management, a pathological lead point was identified in 58% (1331/2294 intussusceptions based on 33 studies with 2193 patients). Benign lesions were the cause of intussusception in 19% of intussusceptions (407/2180 intussusceptions based on 31 articles with 2080 patients). Benign tumors were the cause in 20% of intussusceptions (407/2180 intussusceptions based on 31 articles with 2080 patients). A malignant lead point was found in 25% of intussusceptions (591/2402 intussusceptions based on 36 studies with 2300 patients).

When comparing intussusceptions due to malignant origin based on location (Table 2), enteric intussusceptions were malignant in 9% of enteric intussusceptions (97/1047 enteric intussusceptions based on 29 studies), 46% of ileocolic intussusceptions (93/203 ileocolic intussusceptions based on 15 studies), and 68% of colocolic intussusceptions (204/298 colocolic intussusceptions based on 25 studies).

**TABLE 2:** List of reported malignant pathologies.

Malignant tumors	Colo‐colic	Ileo‐colic	Enteric	Total
Adenocarcinoma	121	33	7	161
Lymphoma	26	19	18	63
Metastasis	4	2	14	20
Metastatic melanoma	4	0	12	16
Melanoma	3	2	7	12
GIST	2	0	11	13
Sarcoma	3	2	2	7
Carcinoid tumor (NET)	2	0	4	6
Plasma cell neoplasm infiltration	0	0	1	1
Carcinomatosis	0	0	1	1
Appendiceal mucocele	1	1	0	2
Malignant Histiocytoma	0	0	1	1
Leukemia	1	0	0	1
Other/not specified	—	—	—	259

The most frequent malignant etiologies reported in our studies were adenocarcinoma, lymphoma, and metastasis. Further details concerning etiologies according to location are described in Table 2. The most frequently reported benign lesions were polyps (92/332 benign lesions), lipomas (85/332), and adenomas (28/332), as depicted in Supporting Information S2.

### 3.4 Management

Operative management was performed in 65% of intussusceptions (1581/2432 intussusceptions, 2330 patients, based on 37 studies). Excluding studies including only surgically operated patients, conservative management was performed in 851/1683 intussusceptions (51%). Data regarding surgical access through open or minimally invasive surgery were available in 14 studies for 778 patients. Most patients (*n* = 564, 72%) underwent open management, whereas 214 (28%) underwent minimally invasive surgery. Data regarding surgical management of the intussusception were heterogenous and poorly reported (available in only 23 studies). En bloc resection without prior reduction was carried out in 676/1063 intussusceptions (64%), reduction and resection were performed in 206/972 (21%), and reduction alone was performed in 66/1063 (6%). Regarding spontaneously reduced intussusceptions, 31 studies reported whether they had negative findings upon exploratory surgery. This was the case in 214/1250 intussusceptions (17%).

Data regarding conservative and surgical treatment can be found in Table 1, whereas Table 3 summarizes the details of surgical management.

**TABLE 3:** Operative management.

First author	Operated patients *n*	En bloc resection *n* (%)	Reduction—resection *n* (%)	Reduction alone *n* (%)	Other type of surgery *n* (%)	Absence of intussusception at surgery *n* (%)	Open *n* (%)	Lap *n* (%)
González‐Carreró [18]	17	NA	NA	NA	NA	0 (0)	NA	NA
Siow [21]	37	34 (92)	0 (0)	3 (8)	0 (0)	0 (0)	17 (46)	20 (54)
Wang [35]	39	21 (53)	18 (47)	0 (0)	0 (0)	0 (0)	NA	NA
Honjo [23]	41	4 (10)	30 (73)	7 (17)	0 (0)	0 (0)	29 (70)	12 (30)
Álvarez‐Bautista [12]	25	23 (92)	0 (0)	1 (4)	1 (4)	0 (0)	NA	NA
Dollinger [16]	12	10 (83)	0 (0)	2 (16)	0 (0)	0 (0)	NA	NA
Kim [46]	77	57 (74)	0 (0)	7 (9)	4 (5)	9 (12)	77 (100)	0 (0)
Gupta [29]	27	14 (52)	11 (41)	0 (0)	0 (0)	2 (7)	NA	NA
Dong [11]	88	69 (78)	NA	0 (0)	0 (0)	0 (0)	40 (46)	48 (54)
Neymark [15]	49	32 (65)	0 (0)	8 (16)	0 (0)	9 (18)	29 (59)	20 (41)
Gupta [30]	38	17 (45)	18 (47)	3 (8)	0 (0)	0 (0)	NA	NA
Zubaidi [2]	21	18 (86)	3 (14)	0 (0)	0 (0)	0 (0)	20 (95)	1 (5)
Yakan [34]	20	5 (25)	9 (45)	6 (30)	0 (0)	0 (0)	20 (100)	0 (0)
Somma [25]	32	20 (63)	7 (22)	3 (9)	0 (0)	2 (6)	NA	NA
Gollub [31]	22	22 (100)	0 (0)	0 (0)	0 (0)	0 (0)	NA	NA
Hu [14]	71	NA	NA	NA	NA	NA	NA	NA
Tabrizian [32]	18	NA	NA	NA	NA	6 (33)	NA	NA
Varban [26]	44	NA	NA	NA	NA	14 (32)	36 (82)	8 (18)
Maconi [47]	3	NA	NA	NA	NA	0 (0)	NA	NA
Goh [43]	60	54 (90)	5 (8)	1 (2)	0 (0)	0 (0)	NA	NA
El Fortia [44]	30	NA	NA	NA	NA	NA	30 (100)	0 (0)
Sundaram [33]	8	NA	NA	NA	NA	0 (0)	NA	NA
Olasky [36]	11	NA	NA	NA	NA	2 (18)	NA	NA
Sandrasegaran [45]	1	1 (100)	0 (0)	0 (0)	0 (0)	0 (0)	NA	NA
Ahn [37]	42	28 (67)	14 (33)	0 (0)	0 (0)	0 (0)	NA	NA
Rea [40]	30	NA	NA	NA	NA	10 (33)	23 (77)	7 (23)
Chang [38]	44	NA	NA	NA	NA	1 (2)	NA	NA
Lindor [48]	77	NA	NA	NA	NA	NA	NA	NA
Tresoldi [39]	20	NA	NA	NA	NA	NA	NA	NA
De Clerck [6]	31	9 (30)	11 (35)	0 (0)	0 (0)	11 (35)	25 (81)	6 (19)
Park [41]	24	NA	NA	NA	NA	0 (0)	NA	NA
Amr [24]	192	123 (64)	16 (8)	19 (10)	11 (6)	23 (12)	148 (77)	44 (33)
Kang [20]	71	42 (59)	17 (24)	1 (1)	2 (3)	9 (13)	30 (42)	41 (58)
Cakir [27]	47	46 (98)	0 (0)	1 (2)	0 (0)	0 (0)	NA	NA
Sun [13]	51	0 (0)	47 (92)	3 (6)	1 (2)	0 (0)	40 (88)	11 (22)
Kim [19]	28	27 (96)	0	1 (4)	0 (0)	0 (0)	NA	NA
Duc [17]	85	NA	NA	NA	NA	NA	NA	NA
Weighted means		74%	51%	12%	5%	21%	78%	43%

Abbreviation: NA, not available.

### 3.5 Treatment Algorithm

**FIGURE 2:**
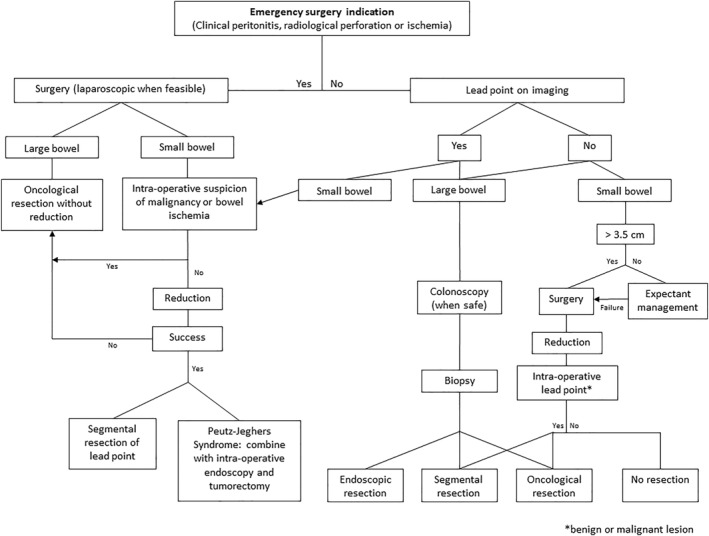
Management algorithm.

Based on the results of this systematic review, we propose a pragmatic treatment algorithm for management of intussusception (Figure 2). Because of high malignancy rates for both ileocolic and colocolic intussusceptions, these types were grouped under “large bowel.”

## 4 Discussion

Adult intussusception is a rare clinical entity, representing 1%–5% of intestinal obstruction [2]. Diagnosis is challenging, as symptoms may be acute or chronic and are often non‐specific [49]. Because of the rarity of this disease, no clinical guidelines currently exist for its management, and randomized‐controlled trials are not feasible within realistic timeframes. As a result, debates continue regarding both surgical and conservative management, especially with the widespread use of CT scans for diagnosis [50]. When surgery is indicated, debate remains about the type of procedure, the necessity of reduction, and whether resection should follow oncological standards.

The primary outcome of interest of this systematic review was the etiology causing the intussusception.

Even though considering a substantial risk of bias in this systematic review, especially due to publication and reporting bias, lack of etiological data in conservatively treated patients, and the predominance of large surgical series, the high percentage of malignancies in colocolic intussusceptions remains striking. Whereas the majority of intussusceptions are enteric and benign, malignancy was identified in 68% of colocolic intussusceptions. These finding are further supported by a multicentric retrospective study from Korea including 77 adult intussusceptions [22]. In their cohort, the authors found that 61.5% of colonic intussusceptions had underlying malignancies and on multivariable analysis, colonic intussusception was found as an independent predictor of malignancy (OR 5.5, 95%CI 1.2‐24.3, *p* = 0.026) along with chronic symptoms (OR 4.6, 95%CI 1.2‐18.1, *p* = 0.031).

Hence, in patients with colocolic intussusception, preoperative workup with colonoscopy and tumor marker dosage is mandatory in the absence of indications for emergency surgery. When emergency surgery is required, oncological resection is the preferred approach. Reduction should not be attempted due to the risk of perforation, intraluminal seeding, or venous tumor dissemination [6, 7].

This is depicted in our clinical guideline outline.

Regarding small bowel intussusception, when the diagnosis is confirmed on imaging, the first step consists of evaluating whether emergency surgery is necessary (e.g., signs of peritonitis, ischemia, or perforation). If not, a preoperative assessment of location and presence of a lead point on imaging should be undertaken.

A large retrospective study of 184 patients [51] comparing CT scans of intussusceptions treated surgically or conservatively found that a discernible lead point was a predictive factor of surgical treatment. Enteric intussusceptions were significantly longer in the surgical group compared to the conservative group (median, 11.3 vs. 3.6 cm, *P* < 0.001).

When a lead point is discovered on pre‐operative imaging or during surgery, and there are no signs of complication (ischemia, perforation, or malignancy), reduction of the enteric intussusception may be attempted as these represent a majority of benign etiologies. We reported 91% of benign etiologies in our systematic review. In case of a failed reduction, further attempts should not be performed, and a “en bloc” resection should be carried out to avoid intestinal damage or perforation. In case of a successful reduction and presence of a lead point, a segmental resection should be carried out. If no lead point is found, resection is not recommended.

In the absence of a lead point in enteric intussusceptions on pre‐operative imaging, studies have shown that short intussusceptions < 3.5 cm can be managed conservatively [40, 52]. This is why it was decided to include this threshold in the present algorithm [16]. However, size of the intussusception was scarcely documented in the selected studies, and therefore not represented. In the absence of a lead point, if the enteric intussusception is 3.5 cm or longer, surgery with attempt of reduction is recommended in order to avoid extensive bowel resection. If the intussusception is under 3.5 cm, we recommend conservative treatment with close clinical and biological follow‐up. This is aligned with the transient characteristic of some enteric intussusceptions without obstruction or lead point identified on CT [36, 42]. In this review, 17% of operated intussusceptions had spontaneously reduced and were not found during surgical exploration. Etiologies of “idiopathic” intussusception without a lead point probably underestimate and overlook etiologies such as hereditary angioedema, inflammatory diseases (Crohn's), or immune conditions (HIV) [53, 54].

When operative management is indicated, surgical access should be evaluated. Laparoscopic access has become increasingly popular in recent years thanks to surgical equipment development and widespread use of minimally invasive techniques. Laparoscopic access can be safely used as an initial access, which is particularly interesting for enteric intussusceptions which may be transient and not require a bowel resection. Treatment of intussusception can be safely performed using a minimally invasive approach; however, it depends on the surgeon's experience. Kang et al. [20] recommend exercising caution when attempting bowel reduction laparoscopically, as handling the bowel with graspers may cause injury, particularly in cases where the tissue is friable.

Several studies did not reveal significant differences between open and laparoscopic groups regarding operative time, whereas describing fewer complications in the laparoscopic group [13, 20, 21]. All studies had low conversion rates 0%–2.4% [13, 20, 21]. Patients in laparoscopic groups had faster recovery with a shorter hospital stay and a faster time to oral intake [13, 20, 21].

A specific group where laparoscopic access is particularly valuable in patients with Peutz–Jeghers syndrome as they are at risk of recurrent intussusception because of the formation of hamartomatous polyps throughout the digestive tract. In their case, when intussusception is surgically confirmed, conversion to a mini‐laparotomy should be undertaken to perform an enterotomy and intraoperative enteroscopy to rule out the presence of multiple polyps or hamartomas. This may avoid repeat surgeries and minimize the risk of short bowel syndrome associated with multiple resections [55].

A systematic review and meta‐analysis on intussusception in adults was published in 2019 [56]. Eligibility criteria were different from the present study. The authors included patients older than 15 years and case series of 10 or more patients. A total of 40 retrospective case series was included (1229 patients). The main findings of this study are in line with the results of the present systematic review. CT was identified as the most accurate preoperative imaging tool for diagnosis, with enteric intussusceptions being the most commonly observed type (pooled rate 49.5%). Their results also found the vast majority of colonic intussusceptions were due to primary adenocarcinoma (pooled rate 78.8%).

The present study has several limitations that need to be mentioned. Existing data is heterogeneous and derived from retrospective studies with inherent limitations. Although the level of evidence supporting the suggested treatment algorithm is low, it intends to serve as a general guide in day‐to‐day practice but should be adapted to the clinical scenario of every patient. There is a risk of selection bias as most of the included series were surgical or radiological cohorts, causing disparities in collected data and making comparison of these studies difficult. The majority of the patients in these studies had undergone surgery, inducing a potential selection bias with possible overestimation of malignancy rates. In that sense, this review might also be impacted by a publication bias: patients treated conservatively were less likely to be published in surgical series, whereas the opposite is true in radiological series. Furthermore, the lack of data on the success and timing of conservative treatment approaches hindered a more in‐depth analysis of different management strategies. Another potential selection bias may be related to the minimum requirement of 20 patients for study eligibility for this systematic review. The aim was to include more robust data, therefore excluding case reports and small case series that constitute most of the literature on this rare clinical entity. Moreover, the quality of data remains suboptimal, as all included studies were retrospective case series with highly heterogeneous data. Despite achieving a median score of 9/10 on the JBI critical appraisal tool for case series studies [8], the variability in the data impeded conducting a meta‐analysis of outcomes. However, the present study incorporates a comprehensive review of existing literature and presents succinctly the best published evidence to date. Prospective multicenter trials are required to refine management strategies, together with surgical studies providing in‐depth description of surgical management and related outcomes. In addition, the suggested pragmatic algorithm proposes standardized management to help clinicians with guidance of this rare pathology in their daily practice.

## 5 Conclusion

In conclusion, this study comprehensively summarizes the existing evidence on intussusception in adults. Current data remain of low quality because of the limited experience and rare presentation of this clinical entity. In the meantime, we propose an algorithm based on the present systematic review and institutional clinical practice to guide decision making when confronted with intussusception. In case of colic intussusception, a high degree of suspicion for an underlying malignancy is warranted, and in that regard, an oncological surgical resection without reduction is recommended. Enteric intussusception should be managed according to the length, clinical presentation, and interoperative findings of lead points.

## Author Contributions

**Sidney Heersche:** Conceptualization, methodology, software, validation, formal analysis, writing – original draft preparation, writing – review and editing. **Jeanne Hirt:** Conceptualization, methodology, software, validation, formal analysis, writing – original draft preparation, writing – review and editing. **Fabio Butti:** Conceptualization, methodology, validation, formal analysis, writing – original draft preparation, writing – review and editing, supervision. **Martin Hübner:** Writing – review and editing. **Dieter Hahnloser:** Writing – review and editing. **Gaëtan‐Romain Joliat:** Conceptualization, methodology, validation, formal analysis, writing – original draft preparation, writing – review and editing, supervision. **Fabian Grass:** Conceptualization, methodology, validation, formal analysis, writing – review and editing.

## Ethics Statement

The authors have nothing to report.

## Conflicts of Interest

The authors declare no conflicts of interest.

## Supporting information


Supporting Information S1

Supporting Information S2
