# Supplementary material for: Intestinal Intussusception in Adults: A Systematic Review
Source: World J Surg. 2025 Aug 19;49(10):2706–16. doi: 10.1002/wjs.70055 (PMC12515024; doi:10.1002/wjs.70055)
Supplement: Supplementary file 2 — Supporting Information S2 [file WJS-49-2706-s001.docx]

**Online appendix**

**Supplementary material**

Table detailing number of benign lesions according to type of intussusception. Displayed from most frequent (top line) to less frequent (bottom line).

| **Benign lesions** | Colo-colic | Ileo-colic | Enteric | Total |
| --- | --- | --- | --- | --- |
| **Polyp** | **17** | **5** | **70** | **92** |
| **Lipoma** | **40** | **12** | **33** | **85** |
| **Adenoma** | **23** | **1** | **4** | **28** |
| Necrosis and hemorrhage | 3 | 7 | 4 | 14 |
| Hamartoma/hemangioma | 1 | 2 | 11 | 14 |
| Infection/inflammation | 5 | 1 | 7 | 13 |
| Meckel | 0 | 0 | 10 | 10 |
| Ischemia | 0 | 2 | 5 | 7 |
| Crohn's disease | 1 | 0 | 5 | 6 |
| Leiomyoma | 2 | 0 | 6 | 8 |
| Submucosal fibrosis | 0 | 0 | 4 | 4 |
| Appendicitis | 0 | 5 | 0 | 5 |
| Anastomosis | 0 | 0 | 4 | 4 |
| Lymphoid hyperplasia | 1 | 1 | 3 | 5 |
| Adhesion | 0 | 0 | 4 | 4 |
| Mucocele | 5 | 2 | 0 | 7 |
| Congenital band | 0 | 0 | 3 | 3 |
| Hematoma | 1 | 0 | 1 | 2 |
| Heterotopic pancreatic tissue | 0 | 0 | 2 | 2 |
| Neurofibroma | 1 | 0 | 2 | 3 |
| Diverticulum | 0 | 1 | 1 | 2 |
| Tuberculosis | 1 | 0 | 1 | 3 |
| Heterotopic gastric tissue | 0 | 0 | 2 | 2 |
| Peritonitis | 0 | 0 | 1 | 1 |
| Granuloma | 0 | 0 | 1 | 1 |
| Lymph node | 0 | 0 | 1 | 1 |
| Foreign body | 1 | 0 | 0 | 1 |
| Celiac disease | 0 | 0 | 1 | 1 |
| Duplication cyst | 0 | 0 | 1 | 1 |
| Oedema | 1 | 0 | 0 | 1 |
| Mesenteric lymphadenitis | 0 | 1 | 0 | 1 |
| Accumulated adipose tissue | 0 | 0 | 1 | 1 |
| *List of reported benign lesions* |  |  |  | **332** |
|  |  |  |  |  |

Quality assessment according to JBI Critical Appraisal Checklist for Case Series [8]

Detailed representation of criteria for JBI quality assessment displayed in green (meeting quality assessment criteria) and red (not meeting quality assessment criteria) color.

**Database Search strategies:**

Detailed representation of search terms according to assessed data sources.

**Pubmed :**

("Intussusception"[MeSH Terms]) AND ("Adult"[Mesh]) AND ("english"[Language]) NOT ("Bariatric Surgery"[Mesh]) NOT ("Rectal Prolapse"[Mesh])

2337 hits

**Web of Science:**

"adult" AND "intussusception" NOT "rectal prolapse" NOT "bariatric surgery" NOT "child" NOT "children" NOT "case report"

865 hits

**Google Scholar:**

"adult" "intestinal intussusception" OR "bowel intussusception" - rectal prolapse - bariatric surgery

295 hits

**Embase/Scopus**

'adult' AND 'intussusception'/exp AND [2004-2024]/py NOT 'rectal prolapse'/exp NOT 'bariatric surgery'/exp NOT 'child'/exp NOT 'case report'

765 hits

**Cochrane**

"intussusception" and "adults"

37 hits

**Total hits: 4299**
